# Influence of metastatic sites and burden on oncological outcomes in patients progressing to metastatic castration resistant prostate cancer

**DOI:** 10.1007/s00345-024-05341-2

**Published:** 2024-11-02

**Authors:** Mike Wenzel, Benedikt Hoeh, Clara Humke, Florestan Koll, Cristina Cano Garcia, Carolin Siech, Thomas Steuber, Markus Graefen, Miriam Traumann, Luis Kluth, Felix K. H. Chun, Philipp Mandel

**Affiliations:** 1 Department of Urology, University Hospital Frankfurt, Goethe University Frankfurt am Main, Frankfurt, Germany; 2 Martini-Klinik Prostate Cancer Center, University Hospital Hamburg-Eppendorf, Hamburg, Germany

**Keywords:** Lymph node, mCRPC, High volume, De Novo, Visceral, Bone

## Abstract

**Purpose:**

Metastatic castration-resistant prostate cancer (mCRPC) patients harbor reduced life expectancy after first-line treatment progression. Currently, no information is available regarding the influence of metastatic sites and osseous burden on progression-free (PFS) and overall survival (OS) of mCRPC patients.

**Methods:**

We relied on the Frankfurt Metastatic Cancer Database of the Prostate (FRAMCAP) database to select patients progressing to mCRPC and stratified them according to lymph node vs. osseous vs. visceral metastatic sites. Moreover, we stratified osseous mCRPC patients regarding the number of metastatic lesions. Endpoints were PFS and OS in uni- and multivariable Cox regression models.

**Results:**

Of 363 patients, 9.4% harbored M1a vs. 78% M1b vs. 12% M1c mCRPC with significantly higher PSA in M1b (9 vs. 22 vs. 8ng/ml). Rates of DeNovo (15% vs. 60% vs. 56%) were significantly lower in the M1a mCRPC group, compared to M1b and M1c (*p* < 0.001). In PFS analyses, a median of 12.7 vs. 10.1 vs. 15.9 months for M1a vs. M1b vs. M1c mCRPC was observed (*p* > 0.05). In multivariable Cox regression models, M1c mCRPC was independently at higher risk for progression (hazard ratio [HR]: 5.93, *p* = 0.048), relative to M1a. Regarding OS, significant differences were observed (*p* = 0.002), with median OS of 58 vs. 42 vs. 25 months for M1a vs. M1b vs. M1c mCRPC and corresponding HRs of 1.54 (*p* = 0.11) and 2.76 (*p* < 0.01). In multivariable models M1c mCRPC was associated with higher risk of death (HR: 3.56, *p* = 0.049), relative to M1a. No differences were observed after stratification according to number of bone lesions (all *p* ≥ 0.05).

**Conclusion:**

M1c mCRPC patients are independently at higher risk for progression and death, while M1a patients harbor best cancer-control outcomes.

**Supplementary Information:**

The online version contains supplementary material available at 10.1007/s00345-024-05341-2.

## Introduction

Androgen deprivation monotherapy (ADT) has been the gold standard treatment for metastatic hormone-sensitive prostate cancer (mHSPC) and metastatic castration-resistant prostate cancer (mCRPC) for many years. Novel additional combination therapies such as docetaxel chemotherapy or androgen signaling receptor inhibitors (ARSI) provide substantially longer overall survival (OS) in mHSPC and time to mCRPC [1–7]. Therefore, most first-line mHSPC treatments aim to extent the time until castration resistant status [8]. However, when status of castration resistance is reached, the metastatic disease becomes a highly lethal disease with reduced life expectancy, compared to mHSPC status [9–11].

For mCRPC patients, several risk factors for poor prognosis have been reported such as initially De Novo metastatic disease, high Eastern Cooperative Oncology Group (ECOG) status, anemia, neutrophil-lymphocyte count ratio, advanced PSA level, or advanced alkaline phosphatase or baseline lactate dehydrogenase [12–18]. In setting of mHSPC, disease volume and burden have been found to be predictive for OS. More specifically, patients with visceral metastases or “high-volume” bone metastases harbored in general worse OS than patients with solely lymph node or osseous metastatic sites of mHSPC disease [19]. However, in the setting of mCRPC, the distinction and influence between lymph node vs. osseous (low vs. high volume according to CHAARTED) vs. visceral metastases on progression-free survival (PFS) and OS outcomes have never been formally addressed.

We addressed this knowledge gap and relied on the FRAMCAP (Frankfurt Metastatic Cancer Database of the Prostate) to investigate cancer-control outcomes such as PFS and OS in mCRPC patients stratified according to lymph node vs. osseous (low vs. high volume) vs. visceral disease at the beginning of mCRPC status. We hypothesized that substantial differences exist between mCRPC patients with M1a vs. M1b vs. M1c disease, which may translate into important PFS- and OS differences.

## Materials and methods

### Study population

After obtaining approval from the local ethics committee (reference number: SUG-5-2024) and adhering to the principles of the Declaration of Helsinki, we retrospectively identified all metastatic prostate cancer patients from the prospective FRAMCAP database. The FRAMCAP database prospectively samples all metastatic prostate cancer patients discussed within a multidisciplinary tumor board since 2014. All patients were treated at the Department of Urology, University Hospital Frankfurt, Germany (*n* = 1127). For analysis, only patients progressing to castration resistant prostate cancer status were included (irrespective of previous mHSPC or non-metastatic HSPC). Patients were excluded if metastatic sites were unknown at status of mCRPC. These criteria yielded 363 mCRPC patients.

### mCRPC definition

mCRPC was defined according to EAU guidelines [20]: Three consecutive rises of PSA values during mHSPC treatment with a PSA level above 2ng/ml and 50% rise above the nadir. Moreover, radiographic progression of two new osseous or one soft tissue metastasis using RECIST (Response Evaluation Criteria in Solid Tumors) were considered as new mCRPC status. Patients were able to undergo conventional imaging for staging or PSMA-PET/CT.

### Statistical analysis

Descriptive statistics comprised frequencies and proportions for categorical variables. Median values and interquartile ranges (IQR) were provided for continuous variables. The Chi-square test assessed the statistical significance of differences in proportions, whereas the t-test and Kruskal-Wallis test were utilized to examine differences in distributions.

For PFS and OS analyses, patients were stratified according to metastatic sites at mCRPC status, namely lymph node (M1a) vs. osseous (M1b, with or without lymph node metastases) vs. visceral metastases (M1c, with or without lymph node and/or bone metastases). Moreover, patients with bone metastases were further stratified according to number of osseous lesions in subgroup analyses.

For all cancer-control outcome analyses, univariable, as well as multivariable Cox regression models were applied. Adjustment in multivariable Cox regression models were performed for age at mCRPC, PSA at mCRPC, Eastern Cooperative Oncology Group (ECOG) status at mCRPC, Gleason score, year of diagnosis, and treatment for first-line mCRPC. OS analyses were additionally adjusted for number of received systemic treatment lines.

All tests were two sided with a level of significance set at *p* < 0.05. R software environment for statistical computing and graphics (version 3.4.3) was used for all analyses.

## Results

Overall, 363 mCRPC patients qualified for final analyses, of which 9.4% (*n* = 34) harbored M1a mCRPC vs. 78% (*n* = 284) M1b mCRPC vs. 12% (*n* = 45) M1c mCRPC (Table 1). Median age at mCRPC was 72 years (IQR 65–78) with a median PSA of 16 ng/ml (IQR: 5–67). Proportions of mCRPC patients with ECOG ≥ 2 were 6.1%. Median follow up was 25 months (IQR 12–43).

**Table 1:** Characteristics of 363 metastatic castration resistant prostate cancer (mCRPC) patients stratified according to metastatic sides

Characteristic	*N*	Overall, *N* = 363^1^	M1a, *N* = 34 (9.4%)^1^	M1b, *N* = 284 (78%)^1^	M1c, *N* = 45 (12%)^1^	*p*-value^2^
**Age at mCRPC**	239	72 (65,78)	74 (67, 79)	72 (65, 78)	71 (65, 78)	0.7
**PSA at mCRPC ng/ml**	256	16 (5, 67)	9 (4, 13)	22 (6, 78)	8 (1, 50)	0.005
**CRPC treatment lines**	363	2.00 (1.00, 3.00)	2.00 (1.00, 4.00)	2.00 (1.00, 3.00)	1.00 (1.00, 2.00)	0.023
**ECOG status at mCRPC**	179					0.5
0		95 (53%)	16 (67%)	71 (51%)	8 (47%)	
1		73 (41%)	7 (29%)	59 (43%)	7 (41%)	
≥ 2		11 (6.1%)	1 (4.2%)	8 (5.8%)	2 (12%)	
**CVD**	241					0.3
0		156 (65%)	19 (79%)	123 (63%)	14 (64%)	
1		85 (35%)	5 (21%)	72 (37%)	8 (36%)	
**Gleason Score**	331					0.2
Gleason 6–7		91 (27%)	10 (33%)	74 (29%)	7 (17%)	
Gleason 8–10		240 (73%)	20 (67%)	185 (71%)	35 (83%)	
**Local therapy RP/RT**	363	167 (46%)	24 (71%)	123 (43%)	20 (44%)	0.01
**De Novo mHSPC**	359	198 (55%)	5 (15%)	169 (60%)	24 (56%)	< 0.001
**Treatment mHSPC**	147					0.061
ADT monotherapy		14 (9.5%)	3 (50%)	10 (8.3%)	1 (5.0%)	
ARSI		75 (51%)	2 (33%)	63 (52%)	10 (50%)	
Docetaxel		42 (29%)	0 (0%)	34 (28%)	8 (40%)	
Triplet therapy		4 (2.7%)	0 (0%)	3 (2.5%)	1 (5.0%)	
Other		12 (8.2%)	1 (17%)	11 (9.1%)	0 (0%)	
**Treatment mCRPC**	363					0.027
ADT monotherapy		25 (6.9%)	2 (5.9%)	16 (5.6%)	7 (16%)	
ARSI		163 (45%)	16 (47%)	134 (47%)	13 (29%)	
Chemotherapy		69 (19%)	7 (21%)	48 (17%)	14 (31%)	
Lu-PSMA		23 (6.3%)	4 (12%)	17 (6.0%)	2 (4.4%)	
Radium		19 (5.2%)	0 (0%)	19 (6.7%)	0 (0%)	
None/Other/NA		64 (18%)	5 (15%)	50 (18%)	9 (20%)	

^1^Median (IQR); n (%)

^2^Kruskal-Wallis rank sum test; Fisher’s exact test; Pearson’s Chi-square test

*Abbreviations* PSA: Prostate-specific antigen, mHSPC: metastatic hormone-sensitive prostate cancer, ECOG: Eastern Cooperative Oncology group, CVD: Cardiovascular disease, RP: Radical prostatectomy, RT: Radiation therapy, Lu-PSMA: Lutetium-Radioligand therapy, NA: Unknown

### mCRPC: M1a vs. M1b vs. M1c

In comparison between mCRPC patients with lymph node vs. bone vs. visceral metastases (Table 1), significant difference in PSA at mCRPC was observed with highest value for bone metastatic patients (9 vs. 22 vs. 8 ng/ml), relative to lymph node and visceral metastatic mCRPC patients. However, no difference in age at mCRPC, proportions of ECOG status ≥ 2 or cardiovascular disease were observed in comparison of all three groups (all *p* ≥ 0.3).

Significant differences in the rates of De Novo mHSPC among the three groups were observed, with the lowest rate in the M1a group (15% vs. 60% vs. 56%, *p* < 0.001). Conversely, rates of local therapy to the prostate were highest in M1a patients, relative to M1b and M1c mCRPC patients (*p* = 0.01). Treatment for mHSPC did not differ between all three groups (*p* > 0.05), while significant differences for mCRPC treatment were observed (*p* = 0.027). Specifically, ARSI was the most frequently used treatment in M1a (47%) and M1b (47%) mCRPC patients, while in M1c mCRPC patients, chemotherapy was the most frequently used treatment (31%) followed by ARSI (29%).

**Fig. 1:**
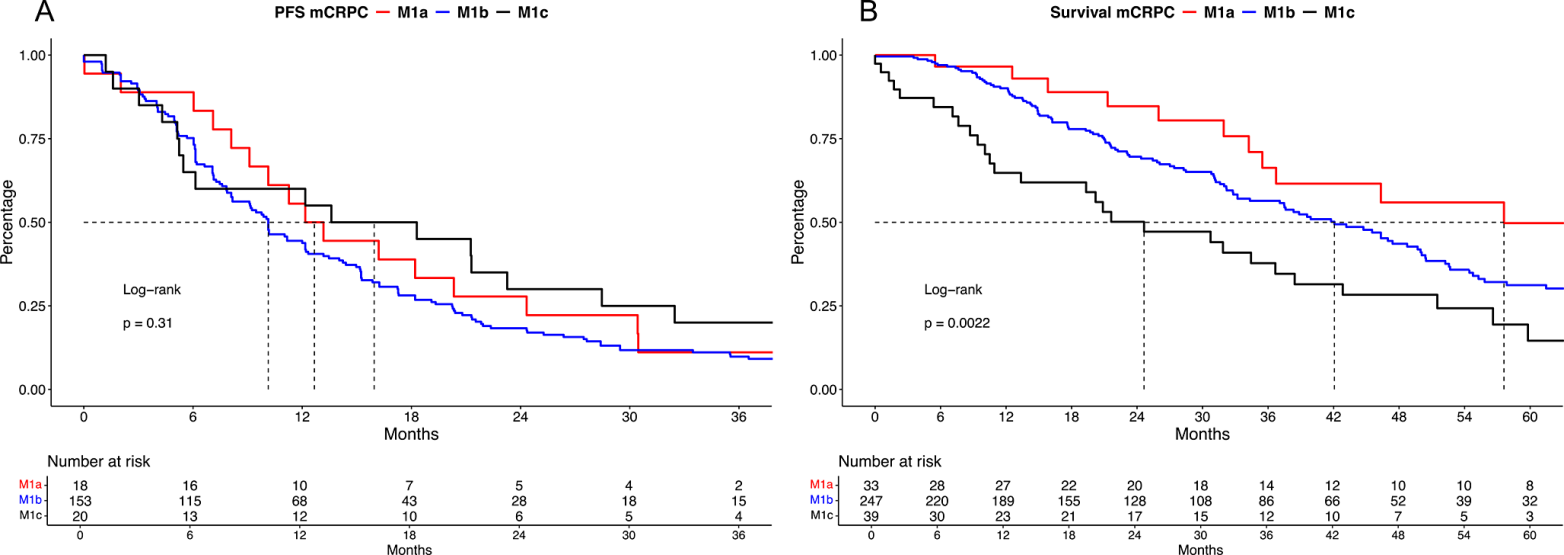
Kaplan Meier curves depicting progression-free survival (PFS, **A**) and overall survival (**B**) in metastatic castration-resistant prostate cancer (mCRPC) patients stratified according to lymph node (M1a) vs. osseous (M1b) vs. visceral (M1c) metastases at mCRPC treatment start

In PFS analyses, no significant differences between all three compared groups were observed (Fig. 1A, *p* = 0.3) with median PFS of 12.7 vs. 10.1 vs. 15.9 months for M1a vs. M1b vs. M1c mCRPC patients. After controlling for patient and tumor characteristics in multivariable Cox regression models, M1c mCRPC patients were independently at higher risk for progression, relative to M1a mCRPC patients (hazard ratio [HR]: 5.93, 95% confidence interval [CI]: 1.02–34.5, *p* = 0.048, Suppl. Table 1A).

In OS analyses, significant differences between the three cohorts were observed (Fig. 1B, *p* = 0.002), with median OS of 58 vs. 42 vs. 25 months for M1a vs. M1b vs. M1c mCRPC and corresponding HRs of 1.54 (CI: 0.90–2.64, *p* = 0.11) and 2.76 (CI: 1.47–5.17 *p* < 0.01). In multivariable Cox regression models after adjustment for patient and tumor characteristics, also M1c mCRPC patients were independently at higher risk of death, relative to M1a mCRPC (HR: 3.56, CI: 1.00-12.65, *p* = 0.049, Suppl. Table 1B).

### Metastatic burden of M1b mCRPC

Of 172 patients with available number of bone metastases, further subgroup analyses were performed. Here, 30% (*n* = 51) M1b patients had 1–3 osseous lesions vs. 70% (*n* = 121) with 4 + lesions.

**Fig. 2:**
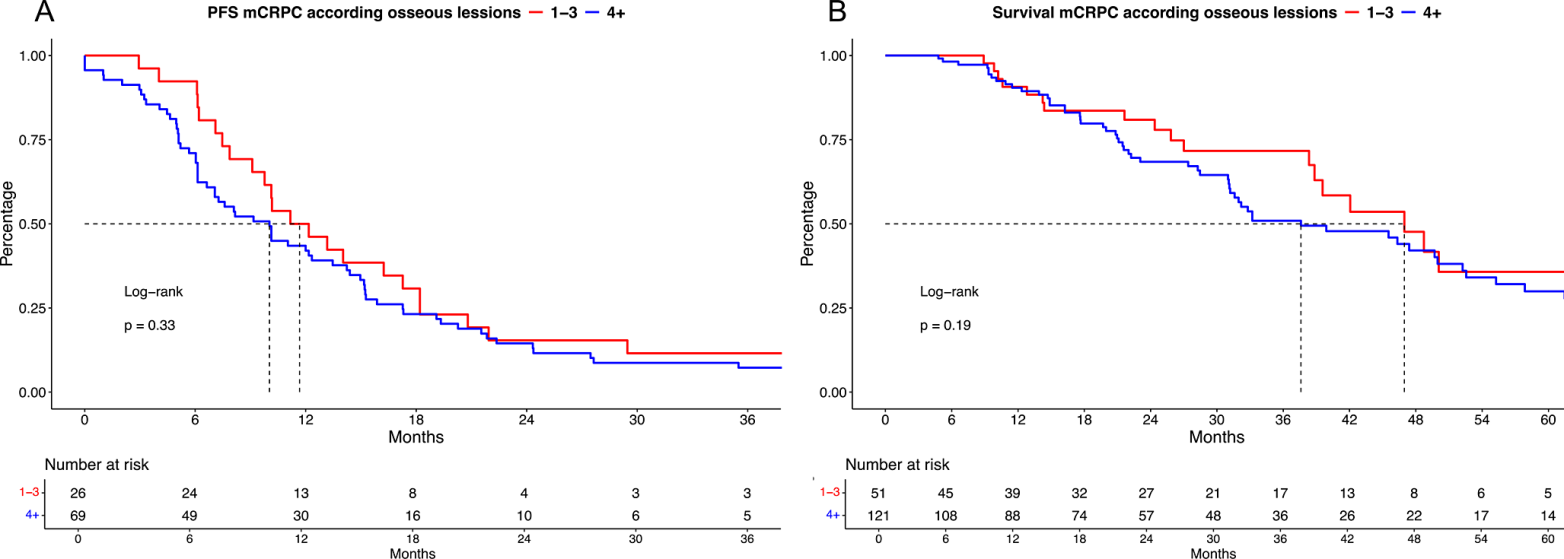
Kaplan Meier curves depicting progression-free survival (PFS, **A**) and overall survival (**B**) in metastatic castration-resistant prostate cancer (mCRPC) patients stratified according to 1–3 vs. 4 + osseous (M1b) metastases at mCRPC treatment start

In PFS outcomes of first-line mCRPC treatment (Fig. 2A), no significant difference was observed with median PFS of 11.7 vs. 10.0 months for 1–3 vs. 4 + bone lesions (*p* = 0.3) with a corresponding HR of 1.26 (CI: 0.79–1.99).

In OS outcome analyses (Fig. 2B), median OS was 47 vs. 38 months for 1–3 vs. 4 + bone lesions (*p* = 0.19), with a corresponding HR of 1.41 (CI: 0.84–2.36). In multivariable Cox regression models, both PFS- and OS analyses did not show significant differences between 1 and 3 vs. 4 + bone lesions (Suppl. Table 1 A-B).

**Fig. 3:**
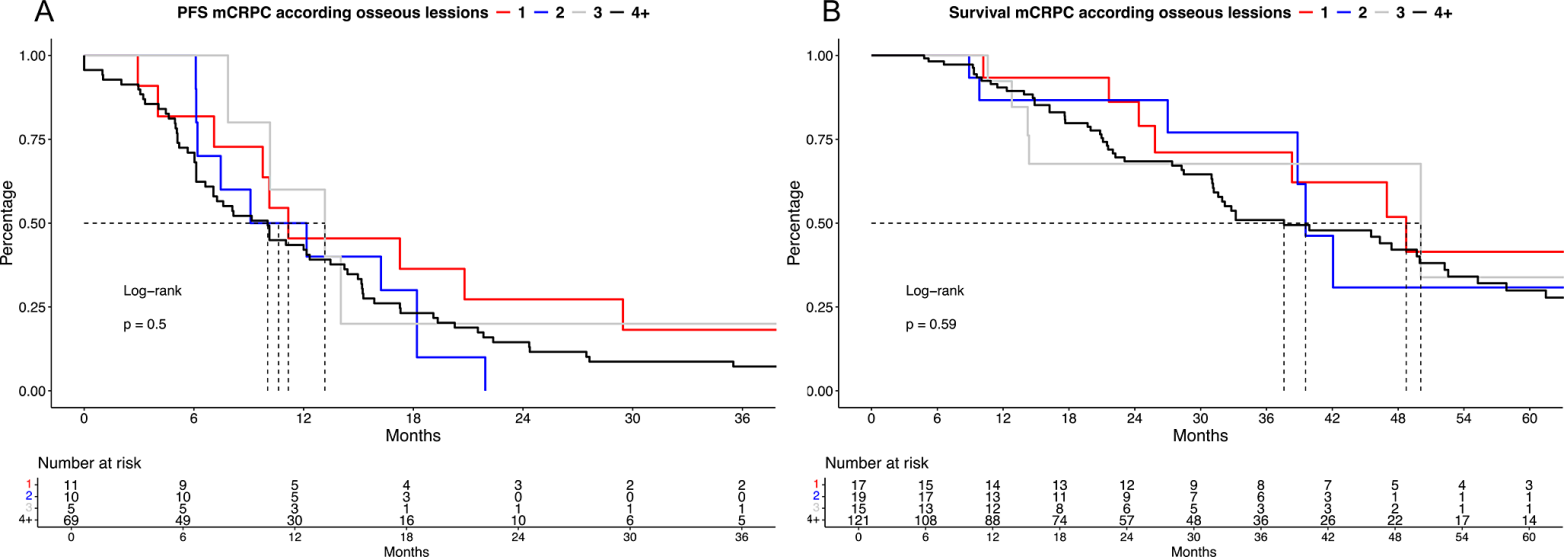
Kaplan Meier curves depicting progression-free survival (PFS, **A**) and overall survival (**B**) in metastatic castration-resistant prostate cancer (mCRPC) patients stratified according to 1 vs. 2. vs. 3 vs. 4 + osseous (M1b) metastases at mCRPC treatment start

In additional subgroup analyses, we further distinguished bone lesions according to 1 vs. 2. vs. 3 vs. 4+ (Fig. 3A-B). Here, we also observed no difference in PFS and OS outcomes.

## Discussion

We hypothesized that substantial differences exist between patients progressing to mCRPC with M1a vs. M1b vs. M1c disease, which may affect cancer-control outcomes such as PFS and OS. We tested this hypothesis within the FRAMCAP database and made several noteworthy observations.

First, we observed significant differences in baseline and tumor characteristics of mCRPC patients stratified according to M1a vs. M1b vs. M1c disease. Specifically, we observed that the vast majority of mCRPC patients harbored bone metastasis (78%), while equally low proportion of patients reaching newly diagnosed mCRPC status harbor only lymph node metastases (9.4%) or visceral metastases (12%). Moreover, median PSA level at mCRPC was significantly higher in bone metastases, relative to M1a and M1c mCRPC patients. Conversely, M1a mCRPC patients harbored lowest rates of initially De Novo and highest rates of local treatment to the prostate. Comparing these observations to previous studies, multiple similarities can be found. For example, rates of visceral metastases are comparable to other previous reports and range between 11 and 27%, depending on the study [21–23]. However, a post-hoc analyses of the COU-AA-301 testing abiraterone after docetaxel chemotherapy in mCRPC patients reported a rate of 29.5% M1c patients with a median PSA of 153 ng/ml [24]. However, it needs to be emphasized that these study-enrolled patients received already one treatment line with progression for mCRPC and are therefore not directly comparable to our real-world study cohort. Moreover, in a recently published cohort of mHSPC patients stratified according to metastatic sites, also M1c patients harbored significantly lower PSA than M1b patients [19]. Finally, the significantly higher rate of local treatment for M1a mCRPC patients may be explained by metachronous recurrent metastatic prostate cancer patients with first presentation of non-regional lymph node metastases or a cytoreductive radical prostatectomy or radiation therapy to the prostate in the setting of low volume De Novo mHSPC [25, 26]. Moreover, some of the included patients may received ADT long-term monotherapy in addition to radiation therapy and developed mCRPC from non-metastatic HSPC.

Second, when cancer-control outcomes between mCRPC patients stratified according to M1a vs. M1b vs. M1c were compared, we observed no difference in univariable analyses PFS. However, after controlling for potentially conflicting patient, tumor and treatment characteristics, M1c mCRPC patients harbored a significant 5.9-fold higher risk of progression, relative to M1a patients. It is of note that it is crucial to adjust these univariable outcomes in multivariable analyses, since baseline tumor characteristics and treatment proportions were substantially different between all three groups (Table 1). Moreover, when OS outcomes were compared, significant median OS differences were observed with favorable OS for M1a and unfavorable OS for M1c mCRPC patients. This OS disadvantage was also present after multivariable adjustment in Cox regression models with a 3.5-fold higher risk of death for M1c mCRPC patients. These findings are in agreement with previous reports [27–29]. For example, an American study of the SEARCH database gathered 494 mCRPC patients of which 16% had visceral metastases [28]. Here, M1c mCRPC patients also exhibited worse median OS, relative to non-M1c mCRPC patients with a median OS of approximately eight months and a 1.8-fold higher risk of death compared to non-M1c mCRPC patients. The median OS reported in this study was substantially shorter than the one reported within the current study (25 months). For its interpretation, it must be emphasized that within the study from Whitney et al., median year of metastatic disease was 2007 [28]. Therefore, the current study provides - despite the higher risk of death for M1c mCRPC patients - also an extended median OS when combination therapies are used within our real-world cohort comparing longitudinal data to those from more historical reports. In consequence, the administration of combination and sequential therapies have almost triplet the life expectancy of patients progressing to M1c mCRPC, relative to M1c mCRPC patients before the era of combination therapies. However, it must be acknowledged that within our trial also M1c mCRPC patients received the lowest median number of systemic treatments, most probability due to rapid disease progression and death.

Finally, when stratification according to CHAARTED criteria regarding bone metastatic burden was performed, we did not observe a significant difference between M1b mCRPC patients with 1–3 vs. 4 + bone lesions and in M1b mCRPC patients with 1 vs. 2 vs. 3 vs. 4 + mCRPC lesions regarding PFS and OS. These observations may indicate that CHAARTED criteria developed for mHSPC distinction may be limited in distinguishing of mCRPC patients due to another tumor pathology. One explanation for this may be the distribution of the metastatic burden in mCRPC setting. For example, within our study, 70% of all M1b mCRPC patients harbored 4 + bone lesions. This hypothesis is also supported by findings of Tablazon et al., in which the authors described an increased mortality risk of 837 mCRPC patients with ten or more bone lesions [30].

Our study has limitations and should be interpreted in the light of its retrospective and single-institutional nature. Additionally, some of our subgroup analyses may lack sufficient sample sizes, potentially impacting the reported outcomes such as the distinction between bone lesions for M1b mCRPC patients. Furthermore, the future interpretation of disease burden due to the widespread use of PSMA-PET/CT for staging and its effect on overall survival remains uncertain. Finally, outcomes may be influenced by other factors such as treatment modalities or sequences or tumor mutations, which we aimed to account for in multivariable adjusted Cox regression models.

## Conclusion

Taken together, the current study provided important information regarding cancer-control outcomes of mCRPC patients stratified according to metastatic sites and burden. Specifically, M1c mCRPC patients are independently at higher risk for progression and death. Distinction of bone lesion according to CHAARTED criteria did not yield sufficient discrimination in M1b mCRPC patients for cancer-control predictions. The findings of the current study should be taken into account when mCRPC patients are counseled regarding life expectancy and discussing treatment options. Moreover, the findings can be used to approximate and schedule next staging appointments.

## Electronic Supplementary Material

Below is the link to the electronic supplementary material.

Supplementary Material 1

## Data Availability

No datasets were generated or analysed during the current study.
