# Supplementary material for: Influence of metastatic sites and burden on oncological outcomes in patients progressing to metastatic castration resistant prostate cancer
Source: World J Urol. 2024 Nov 2;42(1):615. doi: 10.1007/s00345-024-05341-2 (PMC11531415; doi:10.1007/s00345-024-05341-2)
Supplement: Supplementary file 1 — Supplementary Material 1 [file 345_2024_5341_MOESM1_ESM.docx]

|  | Univariable | | | Multivariable | | |
| --- | --- | --- | --- | --- | --- | --- |
| 1. PFS | **HR** | **CI** | **p value** | **HR** | **CI** | **p value** |
| M1a | **Ref.** | **-** | **-** | **Ref.** | **-** | **-** |
| M1b* | 1.13 | 0.70-1.85 | 0.6 | 0.97 | 0.25-3.80 | 0.9 |
| M1c* | 0.79 | 0.42-1.51 | 0.5 | 5.93 | 1.02-34.55 | 0.048 |
| M1b: 1-3 lesions | **Ref.** | **-** | **-** | **Ref.** | **-** | **-** |
| M1b: 4+ lesions* | 1.26 | 0.79-1.99 | 0.3 | 0.99 | 0.43-2.27 | 0.9 |
| 1. OS | **HR** | **CI** | **p value** | **HR** | **CI** | **p value** |
| M1a | **Ref.** | **-** | **-** | **Ref.** | **-** | **-** |
| M1b^+^ | 1.54 | 0.90-2.64 | 0.11 | 0.71 | 0.23-2.18 | 0.6 |
| M1c^+^ | 2.76 | 1.47-5.17 | <0.01 | 3.56 | 1.00-12.65 | 0.049 |
| M1b: 1-3 lesions | **Ref.** | **-** | **-** | **Ref.** | **-** | **-** |
| M1b: 4+ lesions^+^ | 1.41 | 0.84-2.36 | 0.11 | 1.82 | 0.67-4.95 | 0.2 |

Adjustment in multivariable Cox regression models was made for:

*Age at mCRPC, PSA at mCRPC, ECOG status at mCRPC, Gleason Score, year of diagnosis, treatment for first-line mCRPC

^+^ Age at mCRPC, PSA at mCRPC, ECOG status at mCRPC, Gleason Score, year of diagnosis, treatment for first-line mCRPC, amount of systemic treatment lines
